# Alfalfa genomic selection for different stress‐prone growing regions

**DOI:** 10.1002/tpg2.20264

**Published:** 2022-10-12

**Authors:** Paolo Annicchiarico, Nelson Nazzicari, Abdelaziz Bouizgaren, Taoufik Hayek, Meriem Laouar, Monica Cornacchione, Daniel Basigalup, Cristina Monterrubio Martin, Edward Charles Brummer, Luciano Pecetti

**Affiliations:** ^1^ Consiglio per la Ricerca in Agricoltura e l'Analisi dell'Economia Agraria Centro di ricerca Zootecnia e Acquacoltura 29 viale Piacenza Lodi 26900 Italy; ^2^ Institut National de la Recherche Agronomique Centre Régional de Marrakech BP 533 Marrakech 40000 Morocco; ^3^ Institut des Régions Arides Route du Jorf Médenine 4119 Tunisia; ^4^ Ecole Nationale Supérieure Agronomique, Dép. de Productions Végétales. Laboratoire d'Amélioration Intégrative des Productions Végétales (C2711100) Rue Hassen Badi El Harrach 16200 Alger Algérie; ^5^ Instituto Nacional de Tecnología Agropecuaria Estación Experimental Santiago del Estero Jujuy 850 Santiago del Estero 4200 Argentina; ^6^ Instituto Nacional de Tecnología Agropecuaria Estación Experimental Manfredi Ruta Nacional no. 9 km 636 Manfredi Córdoba X5988 Argentina; ^7^ Plant Breeding Center, Dep. of Plant Sciences Univ. of California Davis CA 95616 USA

## Abstract

Alfalfa (*Medicago sativa* L.) selection for stress‐prone regions has high priority for sustainable crop–livestock systems. This study assessed the genomic selection (GS) ability to predict alfalfa breeding values for drought‐prone agricultural sites of Algeria, Morocco, and Argentina; managed‐stress (MS) environments of Italy featuring moderate or intense drought; and one Tunisian site irrigated with moderately saline water. Additional aims were to investigate genotype × environment interaction (GEI) patterns and the effect on GS predictions of three single‐nucleotide polymorphism (SNP) calling procedures, 12 statistical models that exclude or incorporate GEI, and allele dosage information. Our study included 127 genotypes from a Mediterranean reference population originated from three geographically contrasting populations, genotyped via genotyping‐by‐sequencing and phenotyped based on multi‐year biomass dry matter yield of their dense‐planted half‐sib progenies. The GEI was very large, as shown by 27‐fold greater additive genetic variance × environment interaction relative to the additive genetic variance and low genetic correlation for progeny yield responses across environments. The predictive ability of GS (using at least 37,969 SNP markers) exceeded 0.20 for moderate MS (representing Italian stress‐prone sites) and the sites of Algeria and Argentina while being quite low for the Tunisian site and intense MS. Predictions of GS were complicated by rapid linkage disequilibrium decay. The weighted GBLUP model, GEI incorporation into GS models, and SNP calling based on a mock reference genome exhibited a predictive ability advantage for some environments. Our results support the specific breeding for each target region and suggest a positive role for GS in most regions when considering the challenges associated with phenotypic selection.

**Abbreviations**
BLUP: best linear unbiased prediction
GBLUP: genomic best linear unbiased prediction
GBS: genotyping‐by‐sequencing
GEI: genotype × environment interaction
GS: genomic selection
LD: linkage disequilibrium
MAF: minor allele frequency
MS: managed‐stress
PC: principal component
PCA: principal components analysis
QTL: quantitative trait loci
REML: restricted maximum likelihood
RKHS: reproducing kernel Hilbert space
RR‐BLUP: ridge regression best linear unbiased prediction
SNP: single‐nucleotide polymorphism
WG‐BLUP: weighted genomic best linear unbiased prediction

## 1 INTRODUCTION

Alfalfa (alias lucerne, *Medicago sativa* L. subsp. *sativa*) is the most grown perennial forage legume in temperate and Mediterranean‐climate regions (Annicchiarico, Barrett, et al., 2015). It can provide higher protein yield per unit area than any other forage or grain legume (Julier et al., 2017), thereby representing a key asset for securing the farm and regional self‐sufficiency of feed proteins under increasing scarcity of this resource in international markets (Pilorgé & Muel, 2016). Rotations including alfalfa tend to display greater resilience across erratic climatic conditions than rotations excluding this crop (Sanford et al., 2021). In addition, alfalfa may provide important environmental services, including up to 200 kg ha^−1^ of N available for the following crop (with associated energy and climate change mitigation advantages), limitation of soil salinity build‐up by means of deep‐water absorption by its roots, and reduction of soil erosion (Fernandez et al., 2019; Julier et al., 2017). However, the economic sustainability of alfalfa is threatened by its increasing yield gap relative to major cereal crops, which descends from very low rates of genetic yield progress (Annicchiarico, Barrett, et al., 2015). Indeed, alfalfa yield gains derived mainly from improved tolerance to biotic stresses (Lamb et al., 2006). The crop yield progress is limited by several factors that also apply to other outbred perennial forages, such as small breeding investment, long selection cycles, impossibility to select real hybrids or pure lines, and large genotype × environment interaction (GEI) (Annicchiarico, Barrett, et al., 2015). In addition, the yield progress is constrained by a high extent of non‐additive genetic variance due to complementary alleles in the repulsion phase at different loci and intra‐locus allelic interactions allowed for by autotetraploidy (Bingham et al., 1994; Woodfield & Bingham, 1995).

Alfalfa breeding is challenged by the urgent need for more drought‐tolerant cultivars in several growing regions worldwide to cope with lower rainfall and raising evapotranspiration caused by the changing climate and the progressive reduction of irrigation water due to growing water demand for non‐agricultural uses (del Pozo et al., 2019; Polade et al., 2017). A drastic shift from traditional irrigated cropping to rain‐fed cropping or cropping with summer‐suspended or modest supplemental irrigation seems feasible in various regions of North Africa and South America by exploiting exotic genetic resources (Annicchiarico et al., 2011; del Pozo et al., 2017). Salt‐tolerant germplasm is also needed in some regions because of the growing use of saline irrigation water caused by a paucity of good‐quality water and climate change (Paranychianakis & Chartzoulakis, 2005). Cultivars with moderately wide adaptation would be desirable to cope with the wide year‐to‐year climatic variation and the geographic variation for quality and amount of irrigation water. However, commercial alfalfa cultivars and landraces tested across countries of the western Mediterranean basin displayed large GEI for biomass yield that suggested the separate, specific selection for each of three groups of environments definable as severely drought prone, salinity prone, and relatively moisture favorable (Annicchiarico et al., 2011). Indeed, specific germplasm selection for irrigated and rain‐fed environments of Italy produced over two‐fold greater actual yield gains than breeding for wide adaptation (Annicchiarico, 2021).

Modest genetic gains and long selection cycle emphasize the practical importance of exploring selection procedures for alfalfa biomass yield that exploit marker information. Pioneer studies (Musial et al., 2006; Robins et al., 2007) detected quantitative trait loci using 150–200 markers of various types on the limited genetic base represented by F_1_ progenies of a bi‐parental population, which, along with the expected absence of individual markers with high yield effect, limited the practicality of marker‐assisted selection. The occurrence of a large marker × environment interaction for biomass yield was manifest in Robins et al.’s (2007) study. The availability of larger marker numbers enlarged the opportunity to identify quantitative trait loci (QTL) for biomass yield in wider genetic bases, as in Li et al. (2011), McCord et al. (2014), and Ray et al. (2015). The latter study confirmed the complex genetic control of biomass yield under severe drought, based on its association with 25 QTL whose estimated phenotypic effect ranged between 3 and 6%. Likewise, several markers located in different chromosomes were associated with a drought resistance index in a pot experiment by Zhang et al. (2015). The complexity of the drought tolerance trait and the occurrence of large marker × environment interaction for biomass yield were further confirmed in a 1‐yr experiment by Yu (2017), who reported 22 genetic loci associated with yield under drought, of which just a few were also associated with yield under moisture‐favorable conditions. A recent study by Singh et al. (2022) showed the ability of marker‐assisted selection based on 10 QTL to increase biomass yield under deficit irrigation, especially within genetic bases that are genetically similar to those in which the QTL were previously identified.

Genomic selection (GS) combines phenotyping and genotyping data of a genotype sample (training population) representing a target genetic base (breeding population) into a statistical model for prediction of breeding values in future plant selection (Heffner et al., 2009; Meuwissen et al., 2001). This selection strategy is receiving increasing attention for crop yield improvement in alfalfa (Annicchiarico et al., 2021; Hawkins & Yu, 2018) and other crops. It can be more effective than marker‐assisted selection for complex polygenic traits because of the possible high number of genomic regions hosting relevant QTL and the small individual QTL effects (Bernardo & Yu, 2007). Its first applications to alfalfa biomass yield profited from the development of high‐throughput genotyping techniques, such as genotyping‐by‐sequencing (GBS) (Elshire et al., 2011), which can generate thousands of single‐nucleotide polymorphism (SNP) data for a low cost in alfalfa (Li, Wei, et al., 2014) and other crops. Adopting *Ape*kI as the restriction enzyme according to Elshire et al.’s (2011) protocol would be supported by the fact that ∼56% of the alfalfa genome includes repetitive DNA sequences (Long et al., 2022), which this enzyme tends to skip. The potential of GS for alfalfa biomass yield has been investigated based on phenotyping data collected in specific cropping environments. Li et al. (2015) reported a prediction ability (as Pearson's correlation between predicted and observed values) around 0.40 for predictions in a next selection cycle of material selected from parents phenotyped as individual cloned plants under moisture‐favorable conditions. A preliminary assessment of this GS model in terms of actual selection gains was positive for divergent selection of higher‐ and lower‐yielding synthetic populations, and the high‐yield selection produced similar yield gains as the phenotypic selection (Brummer et al., 2019). Jia et al. (2018) reported a prediction ability around 0.25 for biomass yield of replicated clonal material evaluated in China. Medina et al. (2020) reported prediction abilities ranging from 0.27 to 0.30 for annual biomass yield of replicated clonal material evaluated under salt stress. Annicchiarico, Nazzicari, et al. (2015) reported prediction abilities of 0.35 for a Mediterranean breeding population and 0.32 for a Northern‐Italian population evaluated under moisture‐favorable conditions in Italy for genotyped individuals whose breeding value was assessed on their half‐sib progenies phenotyped under relatively dense sward conditions. Such phenotyping conditions had the advantages of (a) predicting additive genetic effects (i.e., the relevant effects for synthetic variety breeding) and (b) representing more faithfully the plant density conditions of actual production environments (Annicchiarico, Barrett, et al., 2015). Andrade et al. (2022) observed a prediction ability for multi‐harvest biomass yield ranging from 0.21 to 0.30. Medina et al. (2020) and Andrade et al. (2022) imputed allele dosage information of this tetraploid species in the GS model, whereas the other studies adopted a diploid SNP calling by pooling the three possible heterozygote classes Aaaa, AAaa, and AAAa into a unique class marked as Aa because of an expectedly insufficient number of reads for attribution to allelic classes for many markers. The study by Annicchiarico, Nazzicari, et al. (2015) suggested distinctly greater predicted yield gains per unit time for GS relative to phenotypic selection based on progeny testing.

**Core Ideas**

- Alfalfa breeding for stress‐prone regions faces large genotype × environment interaction.
- Genomic selection for specific stress‐prone regions may be useful despite low predictive ability.
- Weighted G‐BLUP showed somewhat greater predictive ability than other genomic selection models.

There is no report so far on the GS predictive ability for alfalfa biomass yield in severely drought‐prone environments or across several contrasting cropping environments. Genomic selection models trained on phenotyping data from different test environments can take account of GEI effects and may display better environment‐specific predictions than models based only on data of the single environments (Crossa et al., 2017). Whereas some GEI‐incorporating statistical models represent an extension of models used for data from single environments, other models were proposed specifically for the GEI context to decompose the effect of each marker into a general effect and an environment‐specific effect (Cuevas et al., 2017; Lopez‐Cruz et al., 2015). The adopted GS statistical model among the several ones available (Crossa et al., 2017; Montesinos López et al., 2022; Wang et al., 2018) may affect the model predictive ability for alfalfa biomass yield (Annicchiarico, Nazzicari, et al., 2015; Hawkins & Yu, 2018). In a recent study, a weighted genomic best linear unbiased prediction (WG‐BLUP) GS model exploiting genome‐wide association study results to assign a weight to SNP markers exhibited a sharp prediction accuracy advantage over other popular statistical models (Medina et al., 2021). Another relevant factor that may influence genome‐enabled predictions in alfalfa is the SNP calling procedure for GBS‐generated markers, which may rely on (a) the genome of the model species *Medicago truncatula* (Li, Wei et al., 2014), (b) a mock reference genome based on the linkage disequilibrium of SNP markers observed in a small subset of test genotypes (Puritz et al., 2014), or (c) the alfalfa genome recently sequenced by Chen et al. (2020). Single‐nucleotide polymorphism calling on the alfalfa genome has obvious interest for genome‐wide association studies, but there is little and inconsistent information on its ability to improve the predictive ability of GS models (Annicchiarico et al., 2021). One more factor that may affect GS predictions is the performance of tetraploid or diploid allele dosage (Lara et al., 2019). The former may rely on a statistical criterion for attribution to one of the five classes (AAAA, AAAa, AAaa, Aaaa, aaaa) or may be approximated by computing an A/(A+a) allele ratio that allows for the continuous variation for allele dosage regardless of a definite statistical threshold (de Bem Oliveira et al., 2019).

The main objective of this study was to provide an unprecedented assessment of the ability of GS to predict alfalfa breeding values for biomass yield in agricultural environments of the western Mediterranean basin and Argentina and managed‐stress (MS) environments of Italy. These environments featured a different pattern and extent of drought or, in one environment, irrigation with moderately saline water. The germplasm sample represented a Mediterranean breeding population originated from intercrossing elite, geographically contrasting cultivars selected from material evaluated across several agricultural environments of the western Mediterranean basin (Annicchiarico et al., 2011). Additional aims of the study were to investigate GEI patterns for half‐sib progeny material and their implications for regional selection strategies and to assess the effect on genome‐enabled predictions of different SNP calling procedures, 12 statistical models excluding or incorporating GEI, and the adoption of a diploid allele dosage or a tetraploid allele dosage based on a statistical criterion or the allele ratio.

## 2 MATERIALS AND METHODS

### 2.1 Plant material

The Mediterranean breeding population originated from three elite cultivars that exhibited different adaptation patterns in the study by Annicchiarico et al. (2011): (a) the drought‐tolerant Sardinian landrace Mamuntanas, (b) the moderately salt‐tolerant Moroccan landrace Erfoud 1, and (c) the Australian cultivar SARDI 10, featuring wide adaptation across moisture‐favorable and drought‐prone sites. The autumn dormancy rating of these cultivars ranged from 7 to 10. The cultivars were intercrossed in isolation for two generations as described in Annicchiarico, Nazzicari, et al. (2015). The first intercrossing generation included 70 randomly chosen genotypes from each population. One seed per parent plant was grown to establish the second intercrossing generation. This study included 127 genotypes randomly sorted out of the breeding population, which were genotyped and underwent phenotyping based on their half‐sib progenies in four agricultural environments and in two MS environments. One MS environment hosted the half‐sib progenies of 23 additional genotyped individuals, thereby phenotyping 150 genotypes. These genotypes represented a subset of the 154 genotypes evaluated by Annicchiarico, Nazzicari, et al. (2015) in a short‐term, moisture‐favorable experiment. The current study included the genotypes for which sufficient half‐sib progeny seed was available for establishing replicated trials of densely planted plots in the current experiments. Half‐seed progeny seed was produced in three large isolation cages pollinated by bumble bee (*Bombus terrestris* L.) families, each including three complete crossing blocks of randomized cloned genotypes, pooling the seed harvested over the nine clones of each genotype.

Three test sites that hosted the earlier evaluation of cultivars by Annicchiarico et al. (2011)—Alger, Médenine, and Oued Tessaout—included also the three cultivars that originated the breeding population (Erfoud 1, Mamuntanas, and SARDI 10) besides the half‐sib progenies to verify whether the site‐specific cultivar adaptive responses that emerged in the earlier study could faithfully be reproduced in the current evaluation.

### 2.2 Phenotyping

Phenotyping for biomass dry matter yield was performed in six test environments described in Table 1. Two MS environments that involved intense and moderate summer drought stress, respectively, were set up in Lodi, Northern Italy, using phenotyping platforms that proved capable of reproducing the alfalfa cultivar adaptive responses observed in agricultural environments with contrasting level of drought stress (Annicchiarico & Piano, 2005). Each platform was composed of four large (24.0 m by 1.6 m by 0.8 m deep) bottomless containers in concrete filled with local sandy‐loam soil, covered by a rainout shelter and equipped with a double‐rail irrigation boom. Both MS environments provided, on average, 240 mm of water (split equally into four irrigation) over the period January–April and 90 mm (split into two irrigations) over the period October–December. Moderate MS, whose water available aimed to represent the climatically unfavorable rain‐fed environments of Italy, also received 120 mm (split into two irrigations) from May to mid‐June and 60 mm (in one irrigation) soon after mid‐September, whereas intense MS received no water from May to the end of September. There were three drought‐prone agricultural test sites: the rain‐fed sites of Alger (Algeria) and Santiago del Estero (Northern Argentina) and the site of Oued Tessaout (Morocco), which was flood‐irrigated with water withholding over the three summer months (which were nearly dry). The dry season occurred in winter in the Argentinian site (unlike the Mediterranean climate, North African sites, and the MS environments). The agricultural site of Médenine (Tunisia) was flood‐irrigated during the whole cropping season by water with 9.37 dS m^−1^ electrical conductivity, a value close to the upper bound of the electrical conductivity range of 2–10 dS m^−1^ that defines moderately saline water (Rhoades et al., 1992) but is expected to produce a modest yield depression in a relatively salt‐tolerant crop such as alfalfa (Cornacchione & Suarez, 2015). Geographical, climatic, and soil information is reported in Table 1 for each environment. Mineral fertilization was incorporated into the seed bed prior to transplanting at the rates of 48 kg ha^−1^ of N and 144 kg ha^−1^ of P_2_O_5_ and K_2_O in both MS experiments; 40 kg ha^−1^ of N, 120 kg ha^−1^ of P_2_O_5_, and 100 kg ha^−1^ of K_2_O in Oued Tessaout; and 150 kg ha^−1^ of P_2_O_5_ and K_2_O in Médenine. No mineral fertilization was provided in Alger and Santiago del Estero.

**TABLE 1:** Acronym, major climatic characteristics, experimental layout, biomass dry matter yield assessment procedures, and mean value and coefficient of variation (CV) of additive genetic variance and experimental error for average annual dry matter yield, for six managed‐stress (MS) or agricultural test environments of alfalfa half‐sib progenies

	MS					
Item	Intense drought	Moderate drought	Alger (Algeria)	Oued Tessaout (Morocco)^a^	Santiago del Estero (Argentina)	Médenine (Tunisia)^a^
Environment acronym	MI	MM	Al	Ot	Se	Me
Latitude	45°19ʹ N	45°19ʹ N	36°45ʹ N	31°49ʹ N	28°02ʹ S	33°20ʹ N
Longitude	9°30ʹ E	9°30ʹ E	3°3ʹ E	7°16ʹ W	64°23ʹ W	10°29ʹ E
USDA soil texture class	sandy loam	sandy loam	clay loam	clay loam	silt loam	sandy loam
Average annual water available, mm	330	510	546	739	692	1,900
Average daily mean temperature, °C	14.7	13.7	18.7	19.7	18.9	21.9
Average daily maximum temperature, July–Aug., °C	29.7	32.5	32.8	45.7	35.4	36.4
Experiment establishment	Apr. 2013	Apr. 2012	Dec. 2014	Oct. 2013	Sept. 2015	May 2014
Test half‐sib progenies, *n*	150^b^	127	127	127	127	127
Replicates of the randomized complete block design, *n*	4	3	4	5	4	4
Total plants/harvest plants per plot	36/28	36/28	72/42	72/42	72/42	72/42
Plant density at establishment, no. m^−1^	156	156	100	100	100	100
Total no. of test harvests	6	7	17	13	12	16
Mean no. of test harvests over 12 mo	2.4	3.7	6.6	6.2	9.0	7.4
Harvest assessment period	Nov. 2013– Apr. 2016	July 2012– May 2014	Dec. 2015– June 2018	Oct. 2014– Oct. 2016	Dec. 2015– Mar. 2017	Sept. 2015– Oct. 2017
Average annual yield, t ha^−1^ ^c^	5.66	8.33	7.17	12.53	12.82	18.74
Additive genetic variance CV, %	21.9	26.8	19.3	13.8	19.1	20.5
Experiment error CV, %	30.1	19.5	27.0	15.0	15.0	19.1

^a^ Average irrigation amounts were 587 mm for Oued Tessaout (with 3‐mo suspended irrigation during summer) and 1,785 mm of moderately saline water (9.37 dS m^−1^) for Médenine.

^b^ Of which 127 were common to all other test environments. The reported yield and CV values refer to the common set of 127 half‐sib progenies.

^c^ Least‐significant difference at *P* < .05 for row mean comparison = 1.58.

In all experiments, the plant material (half‐sib families and possible cultivars) was sown in plug trays and then transplanted after 5–6 wk in densely planted microplots. Details of the experiments are reported in Table 1. Compared with agricultural environments, MS environments adopted smaller plot size, fewer plants per plot, and greater plant density that derived from between‐ and within‐row plant spacing at 0.08 m instead of 0.10 m owing to less room available for plant material.

Biomass dry matter yield was measured on a plot basis after oven‐drying at 60 °C for 4 d. It was recorded over at least six harvests that were performed over a period of 16–34 m (Table 1). The harvest assessment period excluded one harvest that preceded the occurrence of serious drought in the MS experiments and Santiago del Estero, all harvests that preceded the first summer drought in Alger and Oued Tessaout, and the first harvest in Médenine.

### 2.3 Analysis of phenotyping data

Because biomass yield was recorded over periods of different length in the test environments, plot data were expressed as average annual yield *Y*_A_ (in t ha^−1^) prior to statistical analyses by dividing the total yield over harvests *Y*_T_ by the harvest assessment period reported in Table 1 expressed in months *A*_P_, through the formula *Y*_A_ = 12 *Y*_T_/*A*_P_. For the common set of 127 half‐sib progenies in each experiment, we (a) verified the presence of genetic variation among half‐sib progenies by ANOVA, (b) estimated components of variance relative to half‐sib progeny (*s*_g_^2^) and experimental error (*s*_e_^2^) by a restricted maximum likelihood (REML) method, (c) estimated the additive genetic variance by the formula *s*_a_^2^ = 4*s*_g_^2^ (Posselt, 2010), and (d) expressed this variance and the experimental error variance as CV through division of their square root values by the environment mean. Other ANOVAs verified the presence of genetic variation (a) for the whole set of 150 half‐sib progenies in the intense MS environment and (b) among the cultivars Erfoud 1, Mamuntanas, and Sardi 10 in the environments of Alger, Médenine, and Oued Tessaout. A combined ANOVA including the factors genotype (here “cultivar”), environment, and block within environment assessed the occurrence of GEI for the three cultivars across these sites.

The following analyses investigated biomass yield responses of 127 half‐sib progenies across the six environments. A combined ANOVA including the fixed factor environment and the random factors half‐sib progeny and block within environment was carried out to compare environments for mean yield and to estimate the variance among half‐sib progenies across environments (*s*_g_^2^) and the half‐sib progeny × environment interaction variance (*s*_ge_^2^) by REML analysis. These variance component values were used to estimate the size of the additive genetic variance across environments *s*_a_^2^ and the additive genetic variance × environment interaction *s*_ae_^2^ by the formulas *s*_a_^2^ = 4*s*_g_^2^ and *s*_ae_^2^ = 4*s*_ge_^2^ (Wricke & Weber, 1986). The similarity of environments for GEI pattern was investigated by pattern analysis in its ordination mode as described by Mungomery et al. (1974) and recommended by DeLacy et al. (1996) for GEI investigations aimed to define selection strategies across a target region. Pattern analysis is a principal components analysis (PCA) performed on a genotype–environment matrix of environment‐standardized yield data (DeLacy et al., 1996). We assessed the extent of GEI for half‐sib progeny yield responses across pairs of environments in terms of genetic correlation according to Yamada (1962), testing each correlation for statistical difference to unity (which was indicative of inconsistent response across environments) and to zero on the ground of confidence intervals computed by multiplying standard errors according to Robertson (1959) by relevant *t* values.

Genome‐enabled predictions were assessed on the ground of yield data of 127 half‐sib progenies for all test environments as well as yield data of 150 half‐sib progenies in the intense MS environment. Best linear unbiased prediction (BLUP) yield values of the half‐sib progenies computed for each environment as described in DeLacy et al. (1996) were standardized to zero mean and unit standard deviation prior to genomic selection modeling of site‐specific adaptive responses, as described by Cuevas et al. (2017) for models accounting for GEI effects. Standardizing the data was beneficial also for modeling genotype mean responses across environments to eliminate the greater effect of higher‐yielding environments on genotype means due to the positive relationship between environment mean yield and within‐environment phenotypic variance (Fox & Rosielle, 1982).

All analyses were performed using SAS (SAS Institute, 2008) statistical software.

### 2.4 DNA isolation, GBS library construction, and sequencing

Procedures for DNA extraction and GBS were described in Annicchiarico, Nazzicari, et al. (2015). Briefly, DNA was isolated from fresh leaf tissues of 127 plant samples (150 in the case of the intense MS environment) by the Wizard Genomic DNA Purification Kit (Promega, A1125) and quantified with a Quant‐iT PicoGreen dsDNA assay kit (P7589, Life Technologies). A library was constructed according to Elshire et al. (2011) using 100 ng of each DNA digested with *Ape*KI (NEB, R0643L) and then ligated to a unique barcoded adapter and a common adapter. An equal volume of the ligated product was pooled and cleaned up with QIAquick PCR purification kit (QIAGEN, 28104) for PCR amplification, mixing 50 ng template DNA with 5 nmol each of the primers and NEB 2X Taq Master Mix (NEB cat. no. M0270S) in a 50‐μl total volume. Amplification was carried out on a thermocycler for 18 cycles with 10 s of denaturation at 98 °C, followed by 30 s of annealing at 65 °C and finally 30 s extension at 72 °C. The library was sequenced in two lanes on Illumina HiSeq 2000 at the Genomic Sequencing and Analysis Facility at the University of Texas at Austin.

### 2.5 Genotype SNP calling procedures, data filtering, and imputation strategies

Single‐nucleotide polymorphism calling for the diploid allele dosage was performed using the dDocent pipeline (Puritz et al., 2014), aligning the raw reads on the following three reference genomes: (a) the diploid *M. truncatula* reference genome, version MedtrA17_4.0 (Tang et al., 2014); (b) the reference genome for autotetraploid alfalfa issued from the genome assembly by Chen et al. (2020), for which we selected the longest copy of each chromosome, thereby collating a 685 Mbp sequence out of the original 2,738 Mbp sequence available; and (c) the mock reference genome assembled using dDocent tools, for which we selected the 30 samples with the largest read count to be used as a base for genome assembly and used dDocent ReferenceOpt.sh and RefMapOpt.sh tools to select the following configuration: SIM = 0.92, K1 = 4, K2 = 4. This resulted in a 14.52 Mbp reference FASTA file comprising 152,790 contigs. Single‐nucleotide polymorphism calling was performed on each of the three genomes. The resulting vcf files were filtered for quality using vcftools (Danecek et al., 2011) with options “–remove‐indels –minQ 30 –non‐ref‐af 0.001 –max‐non‐ref‐af 0.9999 –max‐missing 0.3.” The resulting filtered files were transformed in 0/1/2 SNP matrices and further filtered for minor allele frequency (MAF) >5% and three levels of maximum missing rate per marker (1, 3, and 5%) while satisfying the maximum missing rate threshold per genotype of 10%. Markers with heterozygosity ratio >95% were discarded as well. Missing data points in the resulting SNP matrices were imputed according to the k‐nearest neighbors imputation method (Nazzicari et al., 2016). A PCA performed on 53,169 polymorphic SNP markers of the 127 parent genotypes confirmed that the 30 parents selected as founding samples for the dDocent‐mock reference genome represented well the molecular variation of the whole set of parents (Supplemental Figure S1). This analysis and a subsequent analysis of linkage disequilibrium (LD) was performed on markers issued by the diploid SNP calling on the *M. sativa* genome that were filtered for maximum missing rate per marker (3%) and minimum MAF (5%).

We envisaged two thresholds of minimum reads per marker for allele ratios i.e., 20 and 6. These ratios were lower than those recommended by some authors (e.g., de Bem Oliveira et al., 2019) to limit the disadvantage of low marker number expected from the low sequencing effort of our study. However, Lara et al. (2019) indicated 25 reads per marker as a suitable threshold for GS predictions, and Ferrão et al. (2021) reported negligible differences in GS prediction ability between allele ratios based on minimum reads per marker of 6 and 60 in studies on other tetraploid species.

Single‐nucleotide polymorphism calling for allele ratios and tetraploid genotypes was performed on the *M. sativa* genome starting from the reads already trimmed and aligned by dDocent pipeline. Read counts and quality were obtained using the freeBayes software (Garrison & Marth, 2012) using the “naive” configuration, as per software manual, using options “–haplotype‐length 0 –min‐alternate‐count 1 –min‐alternate‐fraction 0 –pooled‐continuous –report‐monomorphic ‐m 5 ‐q 5.” We then used bcftools software (Danecek et al., 2021) to subset the variants to biallelic SNPs only using options “view ‐m2 ‐M2 –types snps.” We obtained two matrices for counts of reference allele and total depth (reference plus alternate alleles) through a custom R script. In the same step, we filtered on minimum depth thresholds (6 and 20). At this stage it was already possible to obtain the ratio matrices (i.e., two matrices; one per read depth threshold) containing for each SNP and DNA sample the ratio of reference alleles over the total alleles (or a missing point in case of insufficient read depth). To obtain the properly called “genotypes,” we input the count matrices filtered for minimum depth equal to six to *updog* function from R package *updog* (Gerard et al., 2018) with ploidy either set to four (tetraploid) or two (diploid). We then filtered on calling quality removing the calls with an estimated proportion of individuals misclassified in the SNP above 0.05 (i.e., the *prop_mis* value returned by the *updog* function). The four genotype matrices thus obtained (allele ratios with minimum depth of 6 or 20; tetraploid and diploid called genotypes with minimum depth equal to six) were then filtered for minimum MAF at 5% and for maximum missing rate per marker equal to 1, 3, and 5% to reproduce the same filtering steps used for dDocent genotypes. With reference to genotypes called using *updog*, the diploid markers were strictly a superset of the tetraploid ones. We subset the set of diploid markers obtained using *updog* so that they contained the same set of markers. Finally, missing data points were input using the Random Forest Imputation algorithm from the R package missForest (Stekhoven & Buehlmann, 2012).

### 2.6 Analysis of linkage disequilibrium

Square root values of Pearson's correlation (*r* ^2^) between pairs of markers were collected on a chromosome basis. Following Vos et al. (2017), we estimated LD decay focusing on short‐range LD within a 100‐kb window. Collected values were fitted a polynomial curve as described in Marroni et al. (2011). We measured the distance at which the curve crossed three threshold values (i.e., *r* ^2^ = .1, *r* ^2^ = .2, and *r* ^2^ = LD_½_,_90_). The latter, computed as half of the 90% percentile of *r* ^2^ values at short range, is a LD decay estimator more robust toward the percentage of haplotype‐specific SNPs (Vos et al., 2017).

### 2.7 Assessment of population structure

We investigated the need for imputation of population structure in GS models by a discriminant PCA (Yendle & MacFie, 1989). We used the *k‐means* clustering algorithm iteratively for growing values of *k* genotype groups from 1 to 10 and found the optimal number of clusters according to the local minimum of the Bayesian information criterion. The analyses were performed on the output of a PCA performed on SNP data to benefit from its dimensionality reduction. We considered nine scenarios given by the combination of three genomes (dDocent mock reference genome, *M. sativa*, *M. truncatula*) by three levels of maximum missing rate per sample (1, 3, and 5%). For each scenario, we performed a PCA keeping all principal components (PCs), thereby obtaining nine square 127 × 127 matrices. These matrices were then clustered via *k‐means* for various levels of *k*, assessing the corresponding Bayesian information criterion values. The analysis was implemented using R package Adegenet (Jombart & Ahmed, 2011) using the functions *find.clusters()* and *dapc()*.

### 2.8 Construction and assessment of genomic selection models

We tested several whole‐genome regression models, exploring two distinct scenarios: (a) single‐environment models and (b) GEI‐incorporating models. Predictive ability was always computed as Pearson's correlation between observed and predicted phenotypic values using a 10‐fold cross‐validation scheme, repeating the whole process 10 times for numerical stability and reporting results averaged across repetitions.

We tested five single‐environment models: ridge regression BLUP (RR‐BLUP), genomic BLUP (G‐BLUP), BayesCπ and Bayesian reproducing kernel Hilbert space (RKHS) as described by Wang et al. (2018) and Montesinos López et al. (2022), and WG‐BLUP (Medina et al., 2021). Ridge regression BLUP assumes a linear mixed additive model where each marker is assigned an effect as a solution of the following equation:

y=1μ+Zu+ε

where **y** is the vector of observed phenotypes, μ is the mean of **y**, **Z** is the genotype matrix (e.g., {0,1,2} for biallelic SNPs), **u** ∼ N (0, Iσ^2^_u_) is the vector of marker effects, and **ε** is the vector of residuals. The model is solved in a maximum likelihood context estimating the ridge parameter λ = σ^2^_e_/σ^2^_u_ representing the ratio between residual and markers variance. The model was solved using the function *phenoRegressor.rrBLUP* from R package GROAN (Nazzicari & Biscarini, 2018) feeding the SNP matrix as *genotypes* parameter.

BayesCπ is somewhat similar to RR‐BLUP, but the equation is solved in a Bayesian context, whereas weights *u* are sampled from a distribution with two component mixture prior with a point of mass at zero and a Gaussian slab. The model was solved using the function *phenoRegressor.BGLR* from R package GROAN with parameters type = ‘BayesC’, nIter = 10000, and burnIn = 1000.

Genomic BLUP uses genomic relationships to estimate the genetic value of an individual. The model uses a realized kinship additive matrix as covariance between individuals, as follows:

y=1μ+g+ε

with the same notation as RR‐BLUP and *g*∼N(0,**G**σ^2^_g_), where **G** is the covariance (additive kinship) matrix computed according to Astle and Balding (2009). The G‐BLUP model has been solved in a maximum likelihood context using the *phenoRegressor.rrBLUP* function from GROAN package feeding the kinship matrix to *covariances* parameter or in a Bayesian context using function *phenoRegressor.BGLR* with parameters type = ‘RKHS’, nIter = 10000, burnIn = 1000. In the latter case we refer to the model as RKHS hereafter.

Weighted G‐BLUP (Medina et al., 2021) is similar to G‐BLUP, but the kinship matrix is computed weighing SNP markers by the *P* values resulting from an association study. The association scores were computed programmatically inside each cross‐validation cycle on the training set using statgenGWAS R package (van Rossum & Kruijer, 2020). Once the scores were obtained, the G* matrix was computed according to Medina et al. (2021) as:

$$G^{*}={\mathrm{ZDZ}}^{\prime }/\left\{2\left[\Sigma p_{i}\left(1-p_{i}\right)\right]\right\}$$

where **Z** is an identity matrix for the markers, **D** is a diagonal matrix where each element of the diagonal corresponds to SNP weights, and *p*_i_ is the observed MAF of all genotyped individuals. The G* matrix should be used instead of **G** in the G‐BLUP model. Given that at the time of writing no implementation of WG‐BLUP was freely available, we implemented a *phenoRegressor.WGBLUP* function to be used in the GROAN framework. Internally, the function computes G* and solves the model using a RKHS Bayesian approach.

We considered seven GEI‐incorporating models. Four were extensions of G‐BLUP, BayesCπ, RKHS, and WG‐BLUP single‐environment models as mixed linear models in which location was modeled as a fixed factor via an incidence matrix, and GEI was modeled as a random factor via the Kronecker product of the location matrix and the genetic kinship matrix (for G‐BLUP, RKHS, and WG‐BLUP) or the SNP matrix (for BayesCπ). The remaining three models were decomposition methods, namely the LCD model proposed by Lopez‐Cruz et al. (2015) and the CD_u and CD_uf models proposed by Cuevas et al. (2017). The LCD model (called “M×E GBLUP model” in the original paper) is an extension of G‐BLUP with the following:

y=1μ+g0+g1+ε

with *g*0∼*N*(0,**G**_0_σ^2^ *_g_*_0_) and *g*1∼*N*(0,*G*_1_σ^2^ *_g_*_1_). In this context **G**_0_ is equivalent to the standard **G** kinship matrix from G‐BLUP, and **G**_1_ is a block diagonal matrix representing the interaction between the genotypes and each environment. In this model, the main effect (*g*0) allows borrowing information between environments (through the off‐diagonal blocks of **G**_0_), and *g*1 captures environment‐specific effects. Operatively, we implemented the decomposition following the instruction in the Box 3a of Supplemental File S4 of the original paper.

The CD_u model considers genetic effects (u) that can be assessed by the Kronecker product of variance–covariance matrices of genetic correlations between environments and genomic kernels (same as in G‐BLUP). The CD_uf model has the same genetic component as the first one plus an extra component, f, that captures random effects between environments. Operatively, we implemented the models following the scripts reported in APPENDIX A and B of the original paper.

Single‐environment 10‐fold cross‐validation was straightforward. Multi‐environment 10‐fold cross‐validation was implemented in two steps. First, a 10% of the lines were randomly selected, and all the data from those lines were removed from the training set (i.e., all the phenotypic data for all environments were removed). The GEI models were then trained on the remaining 90% of the data, and the predictions were done for each environment separately. The usual rotation between 10‐fold cross‐validations was implemented.

Genomic selection predictions relative to single‐environment models were initially assessed for each environment using 127 genotype samples to compare the tetraploid allele dosage or its approximations provided by allele ratios with the diploid allele dosage. The comparison included the diploid SNP calling restricted to the same markers used for the tetraploid dosage to verify the intrinsic advantage of the latter dosage (otherwise potentially handicapped by low marker number). Predictions were averaged across environments with respect to two scenarios: (a) the best single model adopted consistently across all environments and (b) the best environment‐specific models. The results of this comparison supported the subsequent assessment of single‐environment and GEI‐incorporating models based on the diploid SNP calling of data from the three genomes. For the intense MS environment, which included 23 extra genotypes, we assessed prediction ability values for single‐environment models also for the scenario with 150 parent plants.

## 3 RESULTS

### 3.1 Multi‐environment phenotyping

The ANOVA comparison of environments for average annual yield of 127 half‐sib progenies revealed four groups that reflected the environment differences in average annual water available: (a) the continuously irrigated site of Médenine with outstanding production, (b) Santiago del Estero and Oued Tessaout with moderate yield, (c) Alger and moderate MS with fairly low yield, and (d) intense MS with low yield (Table 1). Within‐environment half‐sib variation was always significant (*P* < .01) and, when expressed as additive genetic variance CV, tended to be particularly large in the moderate MS environment (26.8%) and low in Oued Tessaout (13.8%) (Table 1). Relatively higher values of the experimental error CV were displayed by the three lowest‐yielding sites as well as by the top‐yielding site of Médenine (Table 1). Significant (*P* < .01) genetic variation emerged also for the set of 150 half‐sib progenies in the intense MS environment.

The combined ANOVA of three cultivars indicated significant GEI (*P* < .01) across the three sites of Northern Africa. Mamuntanas was the top‐yielding cultivar in the severely drought‐prone site of Alger (*P* < .05), no difference among cultivars emerged in the moderately stressed site of Oued Tessaout, and SARDI 10 out‐yielded the other cultivars in the moisture‐favorable site of Médenine (*P* < .05) (Supplemental Table S1). These responses agreed with those expected on the ground of prior experiments and the modeling of cultivar responses as a function of site rainfall amounts (Annicchiarico et al., 2011).

Both the variance among 127 half‐sib progenies across environments and the half‐sib progeny × environment interaction variance were different from zero (*P* < .01), but the latter was much larger than the former in the REML analysis (Supplemental Table S2). The additive genetic variance × environment interaction exhibited over 27‐fold larger variance than the additive genetic variance across environments [5.176 vs. 0.188 (t ha^−1^)^2^], revealing very large GEI for genetic effects relevant to synthetic variety breeding. This result was confirmed by genetic correlations between pairs of environments for half‐sib progeny yield responses that are reported in Table 2, which were mostly low and occasionally negative. Inconsistent half‐sib family responses occurred across all pairs of environments on the ground of *r*_g_ values always different from unity at *P* < .01. The three drought‐prone agricultural sites of Alger, Oued Tessaout, and Santiago del Estero exhibited no correlation (*P* > .10), whereas the moisture‐favorable site of Médenine was correlated negatively with Oued Tessaout (*P* < .05). The ability of MS environments to predict yield responses in distant agricultural environments was nil for intense MS and fairly modest for moderate MS, which displayed positive genetic correlation (*P* < .01) only with the moisture‐favorable site of Médenine (*r*_g_ = .44) and the moderately stressed site of Santiago del Estero (*r*_g_ = .39). Intense and moderate MS exhibited fairly low genetic correlation (*r*_g_ = .41; *P* < .01).

**TABLE 2:** Genetic correlation for annual biomass dry matter yield of 127 alfalfa half‐sib progenies across six managed‐stress (MS) or agricultural test environments

				MS	
Environment	Oued Tessaout (Morocco)	Santiago del Estero (Argentina)	Médenine (Tunisia)	Moderate drought	Intense drought
Alger (Algeria)	−.19 NS	.00 NS	.23 NS	−.06 NS	−.06 NS
Oued Tessaout (Morocco)	−	−.22 NS	−.35*	−.29†	−.10 NS
Santiago del Estero (Argentina)		−	.14 NS	.39**	.10 NS
Médenine (Tunisia)			−	.44**	.32†
MS, moderate drought				−	.41**

*Note*. NS, not significant. All correlations are different from unity at *P* < .01.

*Significant at the .05 probability level. **Significant at the .01 probability level. †Significant at the .10 probability level.

**FIGURE 1:**
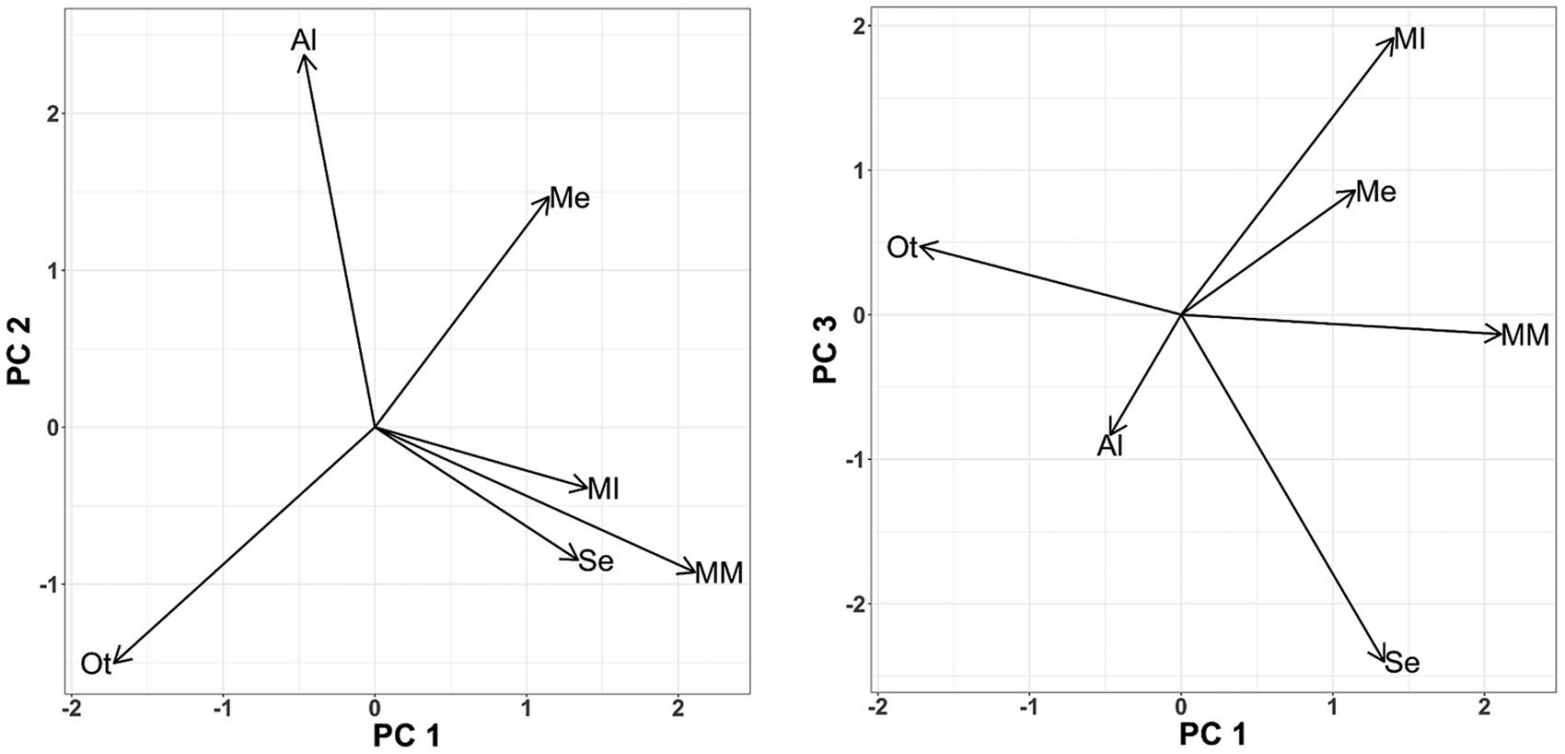
Pattern analysis ordination of six managed‐stress or agricultural test environments as a function of their score on the first three principal component (PC) axes for environment‐standardized average annual biomass dry matter yield of 127 alfalfa half‐sib progenies. Al, Alger; Me, Médenine; MI, intense drought managed stress; MM, moderate drought managed stress; Ot, Oued Tessaout; Se, Santiago del Estero

Pattern analysis results reflected this complex GEI pattern, with the first three PC axes accounting for 25, 19, and 17%, respectively, of the overall GEI variation for half‐sib progeny adaptive responses (Figure 1). Recalling that the inner product between two environment vectors in a two‐dimensional space (as determined by vector lengths and angle between them) is proportional to the phenotypic correlation between environments for half‐sib progeny yield responses (DeLacy et al., 1996), Oued Tessaout, Alger, and Médenine emerged as quite distinct environments for GEI pattern in the space of PC1 and PC2, whereas PC3 tended to separate mainly Santiago del Estero and intense MS (with opposite sign) from the other environments. On the whole, the results of genetic correlation and pattern analysis revealed the high specificity of breeding values for regions of Morocco, Algeria, Argentina, and Italy, as represented by the respective test environments (with Italy represented mainly by moderate MS), which emphasized the investigation of genomic selection opportunities for each individual environment.

### 3.2 Genomic data and genome‐enabled predictions

Next‐generation sequencing produced, on average, 3.0 M reads per genotype sample. The SNP calling for the diploid allele dosage based on the dDocent‐mock reference genome issued more polymorphic markers than the SNP calling on the *M. sativa* genome, which, in turn, produced more markers than using the *M. truncatula* genome, for all thresholds of SNP missing rate per marker (Supplemental Figure S2A). The least stringent threshold of SNP missing data (5%) produced in all cases at least 50,000 polymorphic SNP markers (Supplemental Figure S2A). The least‐favorable configuration in terms of marker number (*M. truncatula* genome with 1% missing data threshold) provided 37,969 markers. The SNP distribution of markers along *M. truncatula* and *M. sativa* genomes is reported in Supplemental Figure S3. In contrast, the SNP calling for the tetraploid allele dosage based on the *M. sativa* genome issued 728 polymorphic SNP markers, whereas allele ratios made available 1,791 and 101 polymorphic SNP markers for the thresholds of 6 and 20 minimum reads per marker, respectively, at the least stringent threshold of 5% SNP missing data (Supplemental Figure S2B).

The measured LD values and the fitted polynomial curve of LD decay are reported in Supplemental Figure S4, and LD decay statistics are reported in Table 3. We observed a quick decay of LD, which crossed all selected thresholds within a few hundred bases. Results were substantially consistent across chromosomes. In particular, LD decayed to *r* ^2^ = .1 within ∼300 bp (with a range of 267 bp for chromosome 7.4 to 331 bp for chromosome 2.4) and to *r* ^2^ = .2 within ∼100 bp (Table 3). The LD_½,90_ value was ∼0.13, with a range of variation from 0.127 to 0.134 (Table 3).

**TABLE 3:** Short‐range linkage disequilibrium (LD) decay within a 100‐kb window

			LD decay		
Chromosome	Median *r* ^2^	LD_½,90_	*r* ^2^ = .1	*r* ^2^ = .2	*r* ^2^ = LD_½,90_
chr1.4	.019	0.127	302	103	216
chr2.4	.021	0.131	331	113	225
chr3.4	.020	0.131	297	101	203
chr4.2	.020	0.134	306	104	202
chr5.2	.021	0.132	295	101	198
chr6.2	.020	0.132	273	93	184
chr7.4	.020	0.132	267	91	180
chr8.1	.020	0.133	274	93	182
Average	.020	0.131	293	100	199

*Note*. Statistics were computed on a chromosome basis. LD decay is reported as the distance in bp where the fitted LD decay polynomial curve crosses the three reported thresholds, rounded to the closest integer. LD_½,90_ is half of the 90% percentile of *r* ^2^ at short range.

The results of the discriminant PCA indicated no need to account for population structure in GS models based on Bayesian information criterion values, which exhibited the lowest value in the absence of genotype clusters (*k* = 1) for all SNP calling genomes and SNP missing data thresholds (Supplemental Table S3).

The preliminary comparison of GS models that neglected or accounted for the tetraploid allele dosage indicated a trend toward higher predictive ability of models based on a diploid allele dosage (Table 4), which was selected for following analyses. This result emerged more sharply for values averaged across environments that were relative to the best single model adopted across all environments (Table 4). An intrinsic advantage of accounting for allele dosage was revealed by the comparison of diploid vs. tetraploid allele dosage based on same number of markers, which revealed at least twofold greater predictive ability of the latter, albeit with low absolute values (Table 4). Predictions based on the tetraploid dosage or its approximation represented by an allele ratio were maximized by the allele ratio based on markers with at least six reads (Table 4), which also maximized the number of polymorphic markers (Supplemental Figure S2B).

**TABLE 4:** Average predictive ability of top‐performing single‐environment genomic selection models implying a tetraploid or a diploid allele dosage across six managed‐stress (MS) or agricultural test environments. Predictive ability relative to the best single model adopted consistently across all environments (PA‐across) or best environment‐specific models (PA‐specific)

Allele dosage	Allele dosage criterion	Genome	PA‐across^a^	Model^b^	PA‐specific^a^
Tetraploid	Statistical^c^	*Medicago sativa*	0.033	RR‐BLUP/0.01	0.091
Tetraploid	Allele ratio, minimum six reads	*Medicago sativa*	0.060	RKHS/0.03	0.110
Tetraploid	Allele ratio, minimum 20 reads	*Medicago sativa*	0.039	RR‐BLUP/0.01	0.082
Diploid	Statistical^c^/markers common to tetraploid dosage	*Medicago sativa*	0.010	RR‐BLUP/0.01	0.045
Diploid	Statistical^d^	*Medicago sativa*	0.088	RKHS/0.05	0.108
Diploid	Statistical^d^	*Medicago truncatula*	0.112	WG‐BLUP/0.03	0.128
Diploid	Statistical^d^	dDocent‐mock reference genome	0.100	WG‐BLUP/0.05	0.116

^a^ As Pearson's correlation between predicted and observed phenotypes in a 10‐fold cross‐validation scheme.

^b^ Statistical model: Bayes, Bayes Cπ; G‐BLUP, genomic best linear unbiased prediction; RKHS, reproducing kernel Hilbert space; RR‐BLUP, ridge regression best linear unbiased prediction; WG‐BLUP, weighted genomic best linear unbiased prediction. Thresholds of single nucleotide polymorphism missing rate per marker: 0.01, 0.03, or 0.05.

^c^ Pipeline for statistical testing: updog.

^d^ Pipeline for statistical testing: dDocent.

The top‐performing single‐environment and GEI‐incorporating GS models for each test environment using a diploid SNP calling are summarized in Table 5. Only the moderate MS environment featured at least one model exceeding 0.25 predictive ability. Best predictions exceeded the values of 0.20 for Alger and Santiago del Estero and 0.10 for Oued Tessaout while being extremely low (<0.10) for Médenine (Table 5). The predictive ability for the intense MS environment was extremely low when considering the common set of 127 genotypes but exceeded 0.10 when considering 150 genotypes (Table 5). We found an advantage of top‐performing GEI‐incorporating models over top‐performing single‐environment models for Alger and Santiago del Estero (with a predictive ability increase >0.035) and a negligible advantage of top‐performing single‐environment models for the other environments (with a predictive ability increase <0.02) (Table 5). Top‐performing GS models were based on the WG‐BLUP statistical model or 1% SNP missing rate per marker in 7 cases out of 13 while showing no predominant SNP calling procedure (Table 5).

**TABLE 5:** Predictive ability (PA) of top‐performing single‐environment and genotype × environment interaction (GEI)‐incorporating genomic selection models for six managed‐stress or agricultural test environments

		Single‐environment model		GEI‐incorporating model	
Environment	No. of genotypes	PA^a^	Model^b^	PA^a^	Model^b^
Alger (Algeria)	127	0.178	MG/Bayes/0.01	0.224	MG/LC/0.01
Oued Tessaout (Morocco)	127	0.122	MS/WG‐BLUP/0.03	0.118	MS/RKHS/0.05
Santiago del Estero (Argentina)	127	0.192	MG/WG‐BLUP/0.01	0.228	MS/WG‐BLUP/0.01
Médenine (Tunisia)	127	0.033	MT/WG‐BLUP/0.01	0.016	MT/CD_uf/0.01
Moderate drought managed stress	127	0.277	MT/WG‐BLUP/0.01	0.266	MG/WG‐BLUP/0.05
Intense drought managed stress	127	0.030	MT/WG‐BLUP/0.03	0.024	MT/G‐BLUP/0.03
Intense drought managed stress	150	0.122	MG/Bayes/0.03	−	−

^a^ As Pearson's correlation between predicted and observed phenotypes in a 10‐fold cross‐validation scheme.

^b^ Diploid SNP calling genome: MG, dDocent‐mock reference genome; MS, *Medicago sativa*; MT, *Medicago truncatula*. Statistical model: Bayes, Bayes Cπ; CD_u, Cuevas et al.’s (2017) decomposition, model u; CD_uf, Cuevas et al.’s (2017) decomposition, model uf; G‐BLUP, genomic best linear unbiased prediction; LC, Lopez‐Cruz et al.’s (2015) decomposition; RKHS, reproducing kernel Hilbert space; RR‐BLUP, ridge regression best linear unbiased prediction; WG‐BLUP, weighted genomic best linear unbiased prediction. Thresholds of SNP missing rate per marker: 0.01, 0.03, or 0.05.

The effects of the diploid SNP calling genome and the statistical model on the maximum predictive ability of single‐environment and GEI‐incorporating GS models are summarized in Table 6, where the predictive ability of the top‐performing models for each combination of SNP calling genome and statistical model are averaged across the six test environments. On average, the best predictions were obtained by the WG‐BLUP model in single‐environment models using *M. truncatula*–issued SNP markers and, averaged across genomes, by the WG‐BLUP model in single‐environment models. However, single‐environment models and GEI‐incorporating Bayesian models performed similarly, based on predictions averaged across models and genomes. The SNP calling genomes ranked in the following order: mock reference genome > *M. truncatula* > *M. sativa* (Table 6). In contrast, top‐performing models averaged across statistical models and test environments indicated no definite advantage of any threshold of SNP missing rate per marker (Supplemental Figure S5).

**TABLE 6:** Effect of the diploid single nucleotide polymorphism (SNP) calling genome and the statistical model on the predictive ability of top‐performing single‐environment and genotype × environment interaction (GEI)‐incorporating genomic selection models

	SNP calling genome			
Statistical model^a^	dDocent‐mock reference genome	*Medicago sativa*	*Medicago truncatula*	Average
Single‐environment models				
Ridge regression best linear unbiased prediction	0.102	0.086	0.090	0.093
Genomic best linear unbiased prediction	0.098	0.091	0.093	0.094
Bayes Cπ	0.097	0.097	0.094	0.096
Reproducing kernel Hilbert space	0.095	0.084	0.090	0.089
Weighted GBLUP	0.111	0.081	0.127	0.107
Average	0.101	0.088	0.099	0.096
GEI‐incorporating Bayesian models				
Genomic best linear unbiased prediction	0.104	0.098	0.091	0.098
Bayes Cπ	0.111	0.088	0.097	0.099
Reproducing kernel Hilbert space	0.100	0.098	0.086	0.095
Weighted GBLUP	0.097	0.076	0.097	0.090
Average	0.103	0.090	0.093	0.095
Other GEI‐incorporating models				
Cuevas et al.’s (2017) decomposition, model u	0.091	0.079	0.093	0.088
Cuevas et al.’s (2017) decomposition, model uf	0.103	0.088	0.091	0.094
Lopez‐Cruz et al.’s (2015) decomposition	0.096	0.089	0.087	0.091
Average	0.097	0.086	0.090	0.091

*Note*. Results averaged across six managed‐stress or agricultural test environments.

^a^ Top‐performing model based on predictive ability as Pearson's correlation between predicted and observed phenotypes in a 10‐fold cross‐validation scheme, using 0.01, 0.03, or 0.05 SNP missing rate per marker. Predictions for 127 common genotypes.

The predictive ability of GS models for genotype mean yield of environment‐standardized yield data was very low, achieving only 0.066 (using mock genome‐derived SNP data and the RR‐BLUP model).

## 4 DISCUSSION

Large GEI for biomass yield emerged for alfalfa cultivars across agricultural environments of different countries within the Western Mediterranean basin (Annicchiarico et al., 2011), MS environments with contrasting level of summer drought (Annicchiarico & Piano, 2005), irrigated and rain‐fed environments of Italy (Annicchiarico, 1992, 2021), and even environments of relatively small target regions such as Tasmania (Pembleton et al., 2010) and the Czech Republic (Hakl et al., 2019). However, the GEI size in the current evaluation of half‐sib progenies, as expressed by 27‐fold greater additive genetic variance × environment interaction relative to the additive genetic variance across environments, was much larger compared with earlier estimates obtained on cultivars. As a relevant comparison, we re‐analyzed the biomass yield data in Annicchiarico et al. (2011) relative to 12 cultivars (including the three parent cultivars of the current breeding population) grown in 10 environments of Algeria, Morocco, Italy, and Tunisia, observing a similar size of cultivar main effects and cultivar × environment interaction components of variance [2.62 vs. 2.54 (t ha^−1^)^2^]. The occurrence of much greater GEI among individual genotypes (as represented by their half‐sib progenies) than among cultivars has great practical importance because genotypes represent the selection unit of breeding programs. This finding may partly be explained by the fact that cultivar variation excludes the variation for adaptation pattern among individuals within cultivar, which proved large when it was evaluated (Annicchiarico, 2007). Large GEI was confirmed by genetic correlations for half‐sib progeny yield responses close to zero not only between the site irrigated with saline water (Médenine) and each of the drought‐prone agricultural sites (Alger, Oued Tessaout, Santiago del Estero) but even between pairs of drought‐prone sites. Although no MS environment of Italy exhibited half‐sib progeny responses very similar to those in agricultural environments of Algeria, Argentina, Morocco, or Tunisia, the fairly low genetic correlation between moderate MS and intense MS environments revealed high GEI even across two levels of drought intensity. The large GEI size supports specific GS or phenotypic selection for each region represented by one test environment (where the moderate MS environment would be representative of rain‐fed Italian environments). The consistency of the current site‐specific ranking of the three parent cultivars in Médenine, Alger, and Oued Tessaout with that observed in a prior study (Annicchiarico et al., 2011) indicates the good repeatability of the cultivar adaptive responses that is a prerequisite for the reliability of GEI analysis results and the selection for specific regions. Alfalfa cultivar GEI patterns proved repeatable also across different Italian environments (Annicchiarico, 2021).

Information on alfalfa adaptive traits that may explain the magnitude of the GEI observed in this study is extremely limited. Although various eco‐physiological studies shed light on mechanisms that contribute to the specific adaptation of alfalfa to severely drought‐prone or moisture‐favorable environments (Annicchiarico et al., 2013; Avice et al., 1997; Kang et al., 2011, 2022; Wissuwa & Smith, 1997) or environments irrigated with saline water (Cornacchione & Suarez, 2015), we are aware of no study that has investigated adaptive traits in relation to different drought stress patterns or different combinations of drought and heat stress. Drought tolerance under summer water deficit after flood irrigation in Oued Tessaout may have resulted from a deep root system that was able to access the stored water. In contrast, a relatively shallow root system may suffice for adaptation to a rainfed Mediterranean‐climate environment such as that of Alger (Ludlow & Muchow, 1990), as confirmed for alfalfa in rainfed Algerian environments by results in Annicchiarico et al. (2013). In addition, Oued Tessaout featured much higher summer temperatures than Alger, whereas Santiago del Estero underwent drought stress in the cool season. Moderate genetic variation for tolerance to high temperatures was reported by Zaka et al. (2016) across the temperature range of 5–35 °C that was distinctly lower than the average daily maximum temperature in summer months at Oued Tessaout (Table 1).

The LD decay within 300 bp for the threshold *r* ^2^ = .1 and within 100 bp for the threshold *r* ^2^ = .2 could be considered quite rapid when compared with values reported in other alfalfa studies. For example, Sakiroglu et al. (2012) observed a value of 500 bp for the threshold *r* ^2^ = .1 in a collection of diploid genotypes belonging to subspecies *caerulea*, *falcata*, or *hemicycla*. On average, LD decayed within 26 Kbp in different alfalfa subpopulations studied by Li, Han, et al. (2014) and within 433 Kbp for a population originated by a factorial mating design among 33 alfalfa genotypes in Andrade et al. (2022) for the threshold *r* ^2^ = .2. Our LD results confirm indirectly that our reference population originated from three geographically contrasting populations was characterized by large genetic diversity, as planned in order to develop GS models of large potential interest for breeding programs. However, the rapid LD decay may complicate GS predictions, which would need large marker numbers to saturate the genome. This condition was hardly satisfied by any SNP calling procedure aimed to account for the tetraploid allele dosage, of which the polymorphic marker number was always below 1,800, when considering that at least 1,000 SNP markers were deemed as necessary for proper genome saturation of alfalfa populations featuring only moderate genetic variation (Li et al., 2011). Greater marker number may have contributed crucially to the predictive ability advantage of the allele ratio based on at least six reads over the other two procedures that took account of the allele dosage. It should be noted that different pipelines and procedures adopted for diploid and tetraploid allele dosages probably contributed to the distinctly greater number of polymorphic markers made available for the diploid dosage. Although insufficient read depth caused by modest sequencing effort limited the exploitation of allele dosage information in the current, genetically broad reference population, one may expect to conveniently exploit the tetraploid allele dosage when adopting greater sequencing effort or reference populations featuring lower genetic diversity. Our comparison of diploid vs. tetraploid allele dosage based on the same set of markers made available by the statistically based attribution of allelic classes confirmed the ability of the latter dosage to improve genome‐enabled predictions, as reported for alfalfa (Medina et al., 2020) and other tetraploid crops (e.g., Lara et al., 2019).

The observed lack of population structure was expected according to the way the reference population was constructed (i.e., the repeated intercrossing of many genotypes from the three populations of origin). This result agrees with earlier findings relative to the same population and to a semi‐dormant reference population obtained by stratified mass selection within a number of Italian landraces and commercial cultivars (Annicchiarico, Nazzicari, et al., 2015).

Even in most favorable situations, the predictive ability of top‐performing GS models was lower than the value of 0.35 reported in Annicchiarico, Nazzicari, et al. (2015) for 154 genotypes sorted out of the same breeding population that were phenotyped for biomass yield under moisture‐favorable conditions in a MS environment. The larger training population sample may have contributed to the greater predictive ability in that single‐environment study, when considering the increase of predictive ability observed for the intense MS environment when analyzing 150 instead of 127 genotypes (0.12 vs. 0.03). High phenotyping costs for this multi‐year, multi‐harvest species prevented the adoption of a larger training population in this multi‐environment study, an evaluation scenario likely to be encountered by any moderately sized breeding program. Higher experimental error and/or lower additive genetic variance may also have contributed to generally lower predictive ability values in this study. We investigated the relationship of drought stress severity with genetic and experimental error variation by estimating their CV under moisture‐favorable conditions in the MS experiment in Annicchiarico, Nazzicari, et al. (2015) with respect to the 127 genotypes that were common to the current study and comparing them with those for moderate MS and intense MS experiments, considering the similarity of these experiments for all aspects (plant density, plot size, phenotyping platform) other than the applied drought stress level. The progressive decrease of predictive ability passing from moisture‐favorable (0.35) to moderate (0.26) and intense managed stress (0.03) was paralleled by a progressive increase of experimental error CV (12.6 vs. 19.5 vs. 30.1%) in the presence of fairly similar additive genetic variance CV (21.8 vs. 26.8 vs. 21.9%). Experimental error CV values in the agricultural environments (Table 1) were always higher than that in the moisture‐favorable MS environment. This finding, and the range of experimental error CV that we found in drought‐prone environments, agree with results for alfalfa in drought‐stress conditions by Singh et al. (2022). Our results suggest that high experimental error, which is a key reason for poor genome‐enabled prediction of crop yield (Montesinos‐López et al., 2022), may hinder the definition of accurate GS models for alfalfa biomass yield for stress environments. However, high experimental error also constrains phenotypic selection for such environments (Blum, 1985).

The observed predictive ability values ranged from moderately low to very low. However, the occurrence of three environments with predictive ability above 0.20 (Alger, Santiago del Estero, and moderate MS) does not compare unfavorably with the values in the range of 0.21 to 0.30 reported by Andrade et al. (2022) for the less challenging case of multi‐harvest biomass yield of alfalfa in a moderately favorable environment in the presence of much slower LD decay, larger training set (177 families), and greater marker number. The actual usefulness of predictive ability values for GS targeted to alfalfa biomass yield depends on the challenges faced by phenotypic selection for this trait as determined by narrow‐sense heritability (*h*_N_^2^) and duration of each selection cycle. The reported values of *h*_N_^2^ for alfalfa biomass yield were in the range of 0.21 to 0.30 (Acharya et al., 2020; Annicchiarico, 2015; Riday & Brummer, 2005), whereas one cycle of half‐sib progeny‐based phenotypic selection may span over 5 yr (inclusive of half‐sib production and first generation of recombination), compared with 1 yr for GS. Based on these premises and assuming *h*_N_^2^ = 0.25 and estimated evaluation costs per genotype (inclusive of VAT) of €68 for GBS‐based GS and €170 for progeny‐based selection, Annicchiarico et al. (2021) identified the predictive ability value of 0.13 as the efficiency threshold for GS relative to phenotypic selection according to predicted yield gains per unit time with same evaluation costs. Such a threshold may actually be somewhat lower for stress‐prone environments, where *h*_N_^2^ values are expected to be decreased by greater experimental error. The 0.13 threshold was exceeded by top‐performing GS models for the moderate MS environment, Alger, and Santiago del Estero, whereas the top‐performing models for Oued Tessaout and intense MS (when based on 150 test genotypes) were not much below. Only Médenine featured predictive ability values of no value for GS. Such a negative result could not be attributed to intrinsically low additive genetic variance or high experimental error, given the intermediate values of their respective CV (Table 1). Our breeding population was not expected to include large variation for tolerance to saline water because Erfoud 1 (the most tolerant of the three contributing cultivars) was characterized by only moderate salt tolerance (Pecetti et al., 2013). The low predictive ability observed for genotype mean yield of environment‐standardized yield data had limited practical importance in our study, given the lack of interest of biomass yield selection for wide adaptation.

Our study confirmed the greater predictive ability of the WG‐BLUP model over other statistical models as put forward for alfalfa biomass yield by Medina et al. (2021), although its current advantage over other models was not as large as that reported by these authors. Our extension of the WG‐BLUP model to incorporate GEI effects confirmed the advantage of this model over other models even under this scenario. Operationally, the adoption of WG‐BLUP is somewhat complicated by the need for a prior association study used to assign SNP marker weights, which has to be performed independently for each cross‐validation run.

We observed a predictive ability advantage of the top‐performing GEI‐incorporating model over the top‐performing single‐environment model only for Alger and Santiago del Estero (with 19–26% relative advantage of the former model; Table 5). The advantage of GEI‐incorporating models over single‐environment models was in the 10–40% range in a set of studies reviewed by Crossa et al. (2017), but these authors anticipated that a sizeable advantage is unlikely to occur for sets of environments featuring very low or negative genetic correlation, such as the current ones. No advantage of GEI incorporation in GS models was reported for other contexts featuring complex GEI patterns (Dawson et al., 2013; Juliana et al., 2020). Our cross‐validation assessment for GEI‐incorporating models hypothesized the prediction of new genotypes in all new locations (wherein validation genotypes are eliminated by all training sets). This scenario is more challenging but seemingly closer to most GS contexts than the alternative scenario wherein validation genotypes for a given environment may be part of a training set in another environment.

The higher number of polymorphic markers provided by the SNP calling based on the dDocent‐mock reference genome probably contributed to its predictive ability advantage over the SNP calling based on *M. truncatula* and *M. sativa* genomes that emerged for results averaged across all statistical models. However, mock reference genome‐based and *M. truncatula* genome‐based SNP callings showed similar value when considering only the best‐performing single‐environment or GEI‐incorporating GS model for each environment (Table 5). A prior comparison of the three SNP calling procedures based on biomass yield under moisture‐favorable conditions and two forage quality traits suggested the similar value for SNP calling of mock reference and *M. truncatula* genomes (Annicchiarico et al., 2021). Single nucleotide polymorphism calling based on the sequenced *M. sativa* genome could exploit only one sequenced genotype so far (Chen et al., 2020), suggesting that greater allelic variation could be captured and exploited once the genome of other genotypes becomes available.

## 5 CONCLUSIONS

This study highlighted the challenge of improving alfalfa biomass yield in stress‐prone growing regions due to large GEI and high experimental errors. The large GEI compels the breeder to select for specific regions, prevents the exploitation of evaluation data from MS or agricultural environments from other regions, and limits the predictive ability gain deriving from GEI‐incorporating GS models over single‐environment models. Genomic selection based on region‐specific models was hindered by low predictive ability values associated with necessarily low training population size and high experimental errors, and it was complicated by rapid LD decay associated with selection within a reference population featuring large genetic diversity. Even under this challenging scenario, GS may be more efficient than phenotypic selection in terms of genetic gain per unit time and unit cost in most of our target regions. The poor efficiency of phenotypic selection for alfalfa biomass yield is supported, inter alia, by the negligible yield improvement observed in the United States in recent decades (Brummer & Casler, 2014). Suitable plant material for comparing GS vs. phenotypic selection in terms of actual yield gains is under generation for all the drought‐prone regions targeted by this study. Even if GS and phenotypic selection displayed similar selection efficiency, GS has the additional potential advantage of allowing to select for other GS‐predictable traits at nearly no extra cost. This can be the case for two key forage quality traits, namely, neutral detergent fiber digestibility and crude protein content, which displayed predictive ability values up to 0.36 and low GEI in this reference population (Annicchiarico et al., 2021; Biazzi et al., 2017). The former trait exhibited moderate predictive ability also in the study by Jia et al. (2018). Greater genotyping effort could increase GS prediction ability by properly exploiting allele dosage information, albeit at a greater genotyping cost (whose impact on the relative efficiency of GS is under verification on other germplasm sets grown in severely drought‐prone environments). The exploitation of more predictive GS models, such as WG‐BLUP in this study, and the perspective availability of greater SNP polymorphism derived from additional sequenced alfalfa genomes, could alleviate the challenge of alfalfa breeding for drought‐prone regions.

## AUTHOR CONTRIBUTIONS

Paolo Annicchiarico: Conceptualization; Formal analysis; Funding acquisition; Investigation; Methodology; Project administration; Resources; Supervision; Visualization; Writing‐original draft. Nelson Nazzicari: Data curation; Formal analysis; Methodology; Software; Visualization; Writing‐review & editing. Abdelaziz Bouizgaren: Investigation; Project administration. Taoufik Hayek: Investigation; Project administration. Meriem Laouar: Investigation; Project administration. Monica Cornacchione: Investigation. Daniel Basigalup: Funding acquisition; Project administration. Cristina Monterrubio Martin: Formal analysis; Visualization. Edward Charles Brummer: Funding acquisition; Project administration; Resources; Supervision; Writing‐review & editing. Luciano Pecetti: Data curation; Investigation; Resources; Supervision; Writing‐review & editing.

## CONFLICT OF INTEREST

The authors declare no conflict of interest.

## Supporting information

Supplemental Table 1. Average annual biomass dry matter yield of three alfalfa cultivars grown in three agricultural environments.

Supplemental Table 2. Analysis of variance, and Restricted Maximum Likelihood estimation of variance components, for average annual biomass dry matter yield of 127 alfalfa half‐sib progenies grown in two managed‐stress and four agricultural environments.

Supplemental Table 3. Bayesian Information Content for growing number of genotype clusters in a Discriminant principal components analysis, for three SNP calling genomes and three thresholds of maximum missing rate per marker (mpm)

Supplemental Fig. 1. Scores on the first two principal component (PC) axes of 127 parent genotypes as a function of 53,169 polymorphic SNP markers in a principal components analysis, with indication of 30 parents selected as founding samples for the dDocent‐mock reference genome (red point = founding sample; grey triangle = other sample)

Supplemental Fig. 2. Number of polymorphic SNP markers as a function of missing rate per marker ranging from 0.01 to 0.05 for diploid SNP calling on the dDocent‐mock reference genome, the *M. truncatula* genome and the *M. sativa* genome (A) and for tetraploid SNP calling on the *M. sativa* genome or its approximation based on allele ratios with at least six or 20 reads per marker (B)

Supplemental Fig. 3. SNP distribution of diploid markers on *M. sativa* (A) and *M. truncatula* (B) genomes. Markers obtained by the dDocent pipeline and filtered for maximum missing rate per marker equal to 3%. The X axis reports the chromosome number and either the selected chromosome copy (for *M. sativa*) or the NCBI GenBank code (for *M. truncatula*). The Y axis reports the SNP position in bp. Each marker is represented by a tile 20,000 bp thick

Supplemental Fig. 4. Plots of linkage disequilibrium (LD) measured as *r^2^* between markers placed within a 100 kb window (close‐range LD). Only a random subset of 3000 data points per chromosome is displayed, to allow for plot readibility. The red line shows the fitted polinomial curve that describes the LD decay

Supplemental Fig. 5. Effect of SNP missing rate per marker ranging from 0.01 to 0.05 on the predictive ability (PA) of top‐performing single‐environment genomic selection models. PA as Pearson's correlation between predicted and observed phenotypes in a 10‐fold cross‐validation scheme. Data averaged across four statistical models and six managed‐stress or agricultural test environments

## Data Availability

The SNP data are available in the NCBI's Sequence Read Archive (SRA) repository at the address: http://www.ncbi.nlm.nih.gov/sra/SRX1421601 in connection with the study by Annicchiarico, Nazzicari et al. (2015).
