# Supplementary material for: Alfalfa genomic selection for different stress‐prone growing regions
Source: Plant Genome. 2022 Oct 12;15(4):e20264. doi: 10.1002/tpg2.20264 (PMC12807203; doi:10.1002/tpg2.20264)
Supplement: Supplementary file 1 [file TPG2-15-e20264-s001.docx]

P. Annicchiarico, N. Nazzicari, A. Bouizgaren, T. Hayek, M. Laouar, M. Cornacchione, D. Basigalup, C. Monterrubio, E.C. Brummer, and L. Pecetti. Alfalfa Genomic Selection for Different Stress-prone Growing Regions.

**Supplemental Table 2. Analysis of variance, and Restricted Maximum Likelihood** **estimation of variance components, for average annual biomass dry matter yield of 127 alfalfa half-sib progenies grown in two managed-stress and four agricultural environments.**

| Source of variation | Degrees of freedom | Mean square | Variance |
| --- | --- | --- | --- |
| Half-sib progeny | 126 | 10.607 ** | 0.047 |
| Environment | 5 | 11114.043 ** | − |
| Block (Environment) | 18 | 139.696 | 1.131 |
| Half-sib progeny × environment interaction | 630 | 9.237 ** | 1.294 |
| Pooled experimental error | 2268 | 4.075 | 4.075 |

*, ** = significant at *P* < 0.05 and *P* < 0.01, respectively; NS, not significant.
