# Supplementary material for: Alfalfa genomic selection for different stress‐prone growing regions
Source: Plant Genome. 2022 Oct 12;15(4):e20264. doi: 10.1002/tpg2.20264 (PMC12807203; doi:10.1002/tpg2.20264)
Supplement: Supplementary file 2 [file TPG2-15-e20264-s007.docx]

P. Annicchiarico, N. Nazzicari, A. Bouizgaren, T. Hayek, M. Laouar, M. Cornacchione, D. Basigalup, C. Monterrubio, E.C. Brummer, and L. Pecetti. Alfalfa Genomic Selection for Different Stress-prone Growing Regions.

**Supplemental Table 1. Average annual biomass dry matter yield of three alfalfa cultivars grown in three agricultural environments.**

| Cultivar | Alger (Algeria) | Oued Tessaout (Morocco) | Médenine (Tunisia) |
| --- | --- | --- | --- |
| Erfoud 1 | 6.322 | 13.723 | 16.040 |
| Mamuntanas | 8.902 | 13.750 | 16.899 |
| SARDI 10 | 6.692 | 13.006 | 20.434 |
| LSD (*P* < 0.05) | 2.147 | NS | 2.788 |

NS, not significant.
