# Supplementary material for: Alfalfa genomic selection for different stress‐prone growing regions
Source: Plant Genome. 2022 Oct 12;15(4):e20264. doi: 10.1002/tpg2.20264 (PMC12807203; doi:10.1002/tpg2.20264)
Supplement: Supplementary file 3 [file TPG2-15-e20264-s003.odt]

P. Annicchiarico, N. Nazzicari, A. Bouizgaren, T. Hayek, M. Laouar, M. Cornacchione, D. Basigalup, C. Monterrubio, E.C. Brummer, and L. Pecetti. Alfalfa Genomic Selection for Different Stress-prone Growing Regions.

Supplemental Table 3. Bayesian Information Content for growing number of genotype clusters (k) in a Discriminant principal components analysis, for three SNP calling genomes and three thresholds of maximum missing rate per marker (mpm)


Number of genotype clusters
Genome
mpm
1
2
3
4
5
6
7
8
9
10
Medicago sativa
0.01
1058
1061
1065
1068
1071
1075
1078
1081
1084
1088
Medicago sativa
0.03
1088
1091
1094
1098
1101
1104
1107
1111
1114
1118
Medicago sativa
0.05
1104
1107
1110
1114
1117
1120
1124
1127
1130
1134
Medicago truncatula
0.01
1043
1046
1049
1053
1056
1059
1063
1066
1069
1073
Medicago truncatula
0.03
1067
1071
1074
1077
1081
1084
1087
1091
1094
1098
Medicago truncatula
0.05
1081
1084
1087
1091
1094
1097
1101
1104
1108
1111
dDocent-mock reference genome
0.01
1109
1113
1116
1119
1122
1126
1129
1133
1136
1139
dDocent-mock reference genome
0.03
1143
1147
1150
1153
1157
1160
1163
1167
1170
1173
dDocent-mock reference genome
0.05
1160
1163
1166
1170
1173
1176
1180
1183
1186
1189
